# Green Tea Extract Increases mRNA Expression of Enzymes Which Influence Epigenetic Marks in Newborn Female Offspring from Undernourished Pregnant Mother

**DOI:** 10.1371/journal.pone.0074559

**Published:** 2013-08-29

**Authors:** Yongkun Sun, Yuuka Mukai, Masato Tanaka, Takeshi Saito, Shin Sato, Masaaki Kurasaki

**Affiliations:** 1 Graduate School of Environmental Science, Hokkaido University, Sapporo, Hokkaido, Japan; 2 Department of Nutrition, Aomori University of Health and Welfare, Aomori, Aomori, Japan; 3 Graduate School of Health Sciences, Hokkaido University, Sapporo, Hokkaido, Japan; 4 Faculty of Health Sciences, Hokkaido University, Sapporo, Hokkaido, Japan; 5 Faculty of Environmental Earth Science, Hokkaido University, Sapporo, Hokkaido, Japan; University of Southampton, United Kingdom

## Abstract

Biochemical and toxicological properties of catechin remain unclear, e.g.; how catechin affects female offspring from undernourished pregnant dams. Here, to elucidate effects of low prenatal protein on female offspring health status, changes of enzymes which modify epigenetic marks related with metabolism in kidneys from newborns were investigated after continuously administering catechin extracted from green tea to lactating maternal rats after pregnant undernourishment. We found that green tea extract intake during lactation up-regulated the activation of AMP-activated protein kinase in young female offspring from protein-restricted dams and modulated the AMP-activated protein kinase pathway in the kidney. This pathway was indicated to be stimulated by SIRT1 gene expression. The feeding of green tea extract to protein-restricted dams during lactation is likely to up-regulate AMP-activated protein kinase activation and may partly lead to alterations of the AMP-activated protein kinase pathway in female offspring kidneys. In addition, energy metabolism in fetal and offspring period with green tea extract administration might be related to enzymes which modify epigenetic marks such as DNA methyltransferase 1 and 3a.

## Introduction

Catechins are water-soluble polyphenols and antioxidants. Tea, red wine, chocolate, and apples are rich in catechins [1]. Epicatechin (EC), epicatechin gallate (ECG), epigallocatechin (EGC) and epigallocatechin gallate (EGCG) are the main catechin substances in tea, all of which are part of the flavonoid family. Flavonoids are secondary plant metabolites. EGCG has the most powerful antioxidant activity of these catechins [2]. The no-observed-adverse-effect level (NOAEL) for heat-sterilized green tea catechins (GTC-H) was 200mg/kg/day for maternal toxicity, and 2000 mg/kg/day for embryo/fetal development [3]. Maternal plasma concentrations of catechins after ingestion of green tea extract (GTE) were about 10 times higher than in rat placentae and 50-100 times higher than in rat fetuses, implying it may have potential benefits for in utero antioxidant protection [4].

Many studies have found tea intake to be associated with lower risks of adult diseases, and it is believed that catechin may be behind some of these health benefits. Catechin eliminates free radicals and decreases cholesterol level preventing cancer development, high blood pressure, arterial sclerosis, thrombosis, heart attacks and brain strokes and aging arrestment [5]. Recently, it has been suggested that EGCG may prevent cognitive impairment in children with fetal alcohol spectrum disorders (FASDs) by inhibiting the activation of oxidative-stress-mediated apoptotic signaling in cognitive deficits associated with FASDs [6].

Protein malnutrition is detrimental at any point in life, but prenatal protein malnutrition has significant lifelong effects. The mother-to-be who suffers from digestive tract dysfunction may become malnourished due to problems with the absorption of nutrients into the body. When pregnant females of various species were given protein malnutrition diets, the offspring were shown to have many deficits including decreased brain weight, increased obesity, and impaired communication within the brain [7]. In addition, maternal protein malnutrition during pregnancy leads to renal morphological and physiological changes [8]. They thought that there was possibility of alterations in the components of the renin-angiotensin system, apoptosis, and DNA methylation. It was indicated that rat offspring exposed to malnutrition during pregnancy may have hypertension and chronic renal disease due to decrease of number of glomeruli. Lloyd et al. [9] pointed out also that a poor diet during pregnancy has been linked to long-term health outcomes for the baby, such as an increased risk of diseases of the heart and kidney. Botden et al. [10] suggested that SIRT1 was an important genetic factor involved in fetal programming during malnutrition, influencing diabetes later in life. SIRT1 is a longevity-related gene and encodes nicotinamide adenine dinucleotide-dependent deacetylase sirtuin-1 and belongs to the SIRT gene family. Among the SIRT gene family, SIRT1 and SIRT2 are considered to play important roles in cell survival, differentiation, metabolism and the cell cycle, and have emerged as candidate therapeutic targets for many human diseases [11]. Furthermore, SIRT1 is considered to play major functions in anti-apoptosis, anti-aging, anti-obesity, caloric restriction and metabolism through deacetylation and modulation of protein functions [12,13], and attenuated oxidative stress-induced apoptosis through p53 deacetylation [14]. In addition, SIRT1 was considered to correlate with DNA methyltransferases (DNMTs) [15]. DNA methylation is catalyzed by DNMTs in mammalian cells. DNMT1 is a large protein (185 kDa) that preferentially methylates hemimethylated DNA. DNMT1 plays important roles on maintenance of methylation patterns, silencing of tumor suppressor genes, and cell survival [16]. On the other hand, DNMT3a is thought to play a role in *de novo* methylation [17]. As effectors of epigenetic marks, how SIRTs and DNMTs affect offspring from undernourished pregnant mothers with and without catechin is still unclear.

Catechins from azuki bean seed coats have been found to attenuate vascular oxidative stress and inflammation and decrease plasma glucose in hypertensive rats [18,19]. However, there are few reports about the effects of catechin in malnutrition during pregnancy [20,21]. Nevertheless, biochemical and toxicological properties of catechins remain unclear. Especially, how catechins affect female offspring from undernourished pregnant mothers. Here, we studied mRNA changes of enzymes which modify epigenetic marks related with energy metabolism in kidneys from offspring of rats that were undernourished during pregnancy and administered catechin extracted from green tea to lactating rats.

## Materials and Methods

### Animals and experimental procedure

All animal experiments were only performed in Aomori University of Health and Welfare. The animal experiments were specifically approved with Animal Experimentation Committee of Aomori University of Health and Welfare.

**Figure 1:**
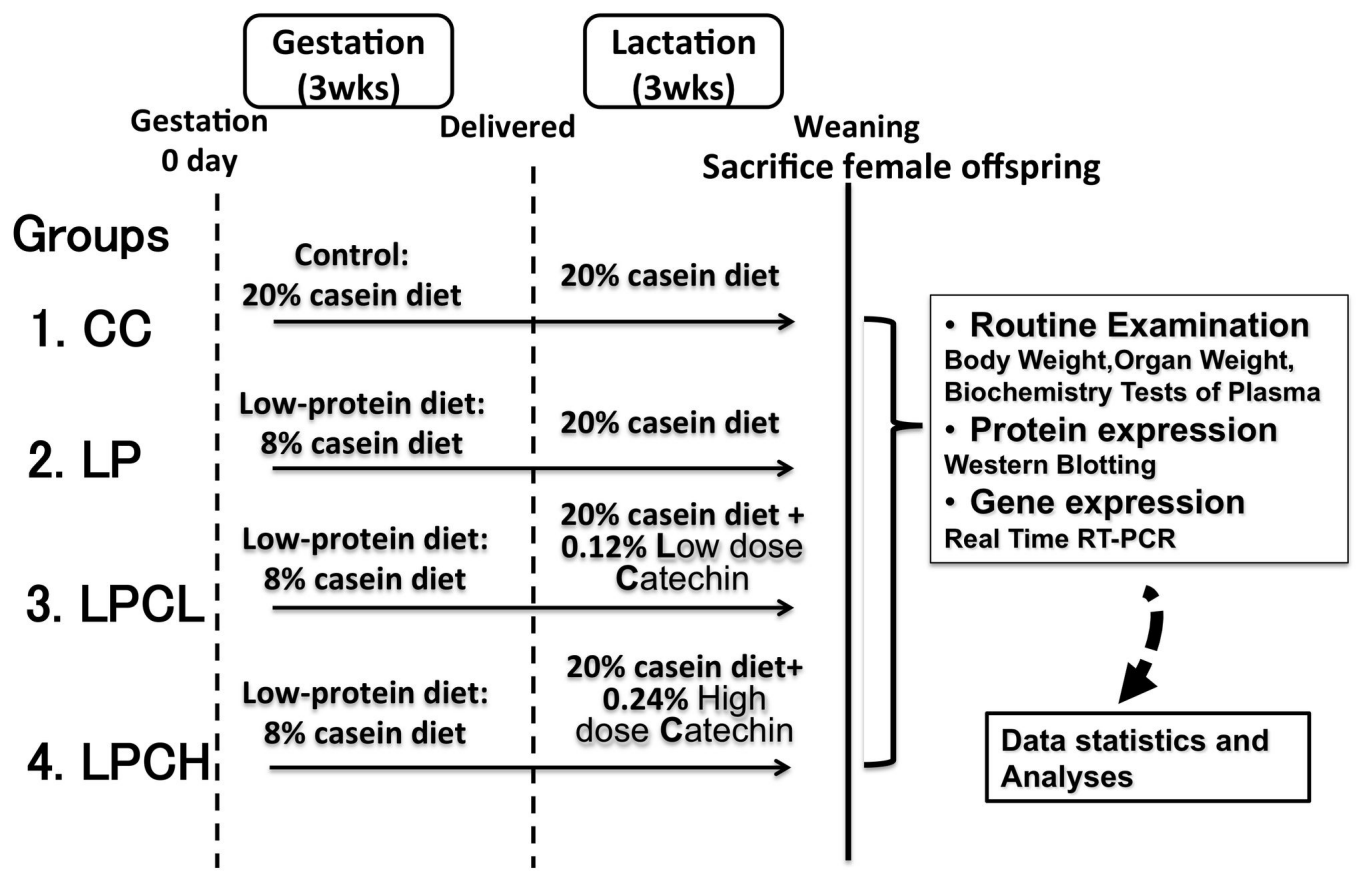
Schematic diagram of experimental design. Pregnant Wistar rats were fed control (20% casein) and low-protein (8% casein) diets during gestation. During lactation, each dam received a control or 0.12% or 0.24% green tea derived catechin-containing control diet.

Eight-week-old virgin female Wistar rats were obtained from Charles River Laboratories Japan Inc. (Yokohama, Japan). The rats were accommodated at a constant temperature of 23 ± 1°C under a 12-h light/dark cycle with access to food and tap water *ad libitum*. A vaginal impedance reader (MK-10C; Muromachi Kikai Co. Ltd., Osaka, Japan) was used to determine whether the female rats were in the appropriate stage of the estrus cycle for mating. This procedure was routinely performed in the afternoon, and a reading of >3 kΩ indicated that the females were in proestrus and presumably in estrus. One female was mated overnight with one male. The next morning, the presence of a vaginal plug indicated successful mating and was documented as day 0 of gestation. Pregnant rats weighing 258-349 g were randomly divided into 4 groups (with 4-8 individuals). Each treatment in the groups is shown in Figure 1 and as follows: CC, control diet during gestation and lactation; LP, low-protein diet during gestation and control diet during lactation; LPCL, low-protein diet during gestation and 0.12% low dose GTE-containing control diet during lactation; LPCH, low-protein diet during gestation and 0.24% high dose GTE-containing control diet during lactation. The no-observed-adverse-effect level in a 2-generation reproductive toxicity study in rats was equivalent to 200 mg/kg/day EGCG preparation [22], which is greater than 160 mg/kg/day EGCG in the 0.24% GTE-containing diet used in our experiment. Moreover, malignant stroke-prone spontaneously hypertensive rats fed 0.5% GTE exhibit delayed stroke onset [23]. Therefore, the amount of catechin used in our study is likely to be pharmacological levels.

The diets were standard commercial laboratory diets (MF diet; Oriental Yeast, Tokyo, Japan) and isocaloric. The composition of the low protein diet was previously described [24]. Dams had access to each food and tap water *ad libitum*. At postnatal day 4, six female pups per dam were randomly selected to ensure adequate nutrition during lactation. At weaning (week 3), the pups were separated from each group and weighed. Then, pentobarbital anesthesia was induced and blood samples were collected, then the livers, kidneys, and heart were removed immediately and weighed. The kidneys were stored at -80°C for the evaluation of mRNA and protein expressions.

### Blood chemistry

Plasma samples were separated by centrifugation at 800 × g for 10 min at 4°C and total cholesterol (T-cho), blood urea nitrogen (BUN), creatinine (CRE), Na^+^ and K^+^ levels were determined using an autoanalyzer for blood chemistry (Fuji, Dry-Chem3500V; Fuji Film, Tokyo, Japan). Insulin was measured using a rat insulin enzymelinked immunosorbent assay (ELISA) Kit (TMB; AKRIN-010T, Shibayagi, Gunma, Japan).

### Western blotting

The kidneys were homogenized in homogenizing buffer (50 mM N-2-hydroxyethylpiperazine-N-2-ethanesulphonic acid [HEPES], 150 mM NaCl, 1 mM dithiothreitol, and 0.5% (v/v) Tween-20; pH 7.4) containing protease inhibitor cocktail tablets (Roche Applied Science, Indianapolis, IN, USA). The homogenates were centrifuged at 5,000× g for 45 min at 4°C. Supernatants were collected, and the protein concentration of the obtained homogenate was measured by Bradford assay (Protein Assay, BIO-RAD, USA) [25]. The homogenate was then centrifuged at 15,000 g for 15 min at 4°C, and the supernatant was transferred to a fresh tube. Tubes were heated for 5 min at 100°C, and 0.1% BPB-glycerol was added. Proteins in the tissue supernatants (20–30 mg) were separated by SDS-PAGE (12.5% and 5%-20% e-PAGEL, ATTO, Japan). Biotinylated protein molecular weight markers (M & S TechnoSystems, Japan) or Odyssey protein molecular weight markers (LI-COR Bioscience, USA) were used as protein standards.

Proteins were then electrophoretically transferred onto a nitrocellulose membrane (Bio-rad, USA) with blotting buffer that contained 48 mM Tris buffer, 39 mM glycine, 0.02% SDS, and 10% methanol, or by using the iBlot transfer system (Invitrogen, USA). The nitrocellulose membrane was incubated overnight at 4°C, in a 5% blocking solution, containing 40 mM Tris–HCl buffer (pH 7.4), 0.9% NaCl, 0.3% Tween 20, and 5% blocking reagent, or by using ODYSSEY blocking buffer (M & S TechnoSystems, Japan). The membrane was washed twice with 40 mM Tris–HCl buffer (pH 7.4), 0.9% NaCl, and 0.3% Tween 20, and then exposed to the diluted primary antibody. AMP-activated protein kinase (AMPK) antibody, Thr172-phosphorylated AMPK antibody, endothelial isoform of nitric oxide synthase (eNOS) antibody, phosphorylated eNOS antibody, p53 antibody (Cell Signaling Technology, USA), and β-actin antibody (Abcam, Japan) were incubated with the blot in a 1% blocking solution that contained 40 mM Tris–HCl buffer (pH7.4), 0.9% NaCl, 0.3% Tween 20, and 1% blocking reagent or ODYSSEY Blocking Buffer. Again, the membrane was washed for 3 times for 3 min in 40 mM Tris–HCl buffer (pH 7.4), 0.9% NaCl, 0.3% Tween 20, and then exposed to the secondary antibody: Anti-Rabbit IgG IRDye 680 or Anti-Mouse IgG IRDye 800 (M & S Techno Systems, Japan), diluted 1500 times in 1% blocking solution. Finally, the membrane was washed 3 times for 3 min in 40 mM Tris–HCl buffer (pH 7.4), 0.9% NaCl, and 0.3% Tween 20. Protein bands were quantitated with Odyssey Infrared Imaging System (M & S Techno Systems, Japan).

### mRNA expression by real-time reverse transcription-polymerase chain reaction (Real-time RT-PCR)

Total RNA of kidney tissue of each offspring was prepared using the SV Total RNA Isolation kit. The PCR primers of DNMT1, DNMT3a, DNMT3b, SIRT1, SIRT2 and β-actin were synthesized according to the DNA sequence as described in our previous study [26]. The real-time RT-PCR condition was executed according to the Rotor-Gene SYBR Green RT-PCR Kit instructions. Briefly, the reverse transcription reaction was carried out at 55°C for 10 min, and subsequently 40 PCR cycles were executed as shown next: initial activation step; 95°C for 5 min, denaturation; 95°C for 5 sec, annealing and extension step; 60°C for 10 sec. β-actin was chosen as an internal control. PCR products were real-time monitored using software of Rotor-Gene Q (USA). Real-time specificity and identity were verified by melting curve analysis of the real-time PCR products. Furthermore to improve accuracy of RNA expression in one step real-time RT-PCR, standard curves were always used for calculating relative copies number of target gene and β-actin on basis of Ct values. Relative values of target gene expression in LP, LPCL and LPCH groups were obtained from ratio of copy number of target genes/β-actin in comparison with that in control group. These values were defined as relative values of mRNA expression for each target gene.

### Statistical analyses

All values are presented as mean ± S.E.M. and treatment groups were compared with one-way analysis of variance (ANOVA), followed by the Fisher’s test.

## Results

### Food intake, offspring body and organ weight

The levels of food intake per day during lactation at PD 0–2 were 16.2 ± 2.4 g, 19.4 ± 2.7 g, 15.6 ± 1.0 g, and 15.2 ± 4.2 g (mean ± SD) in the CC, LP, LPCL, and LPCH groups, respectively; at PD 10–12 they were 51.2 ± 2.4 g, 53.0 ± 3.2 g, 47.7 ± 2.8 g, and 46.9 ± 4.1 g, respectively; and at PD 20–21 they were 82.5 ± 20.1 g, 91.6 ± 11.1 g, 77.0 ± 3.4 g, and 78.3 ± 11.1 g, respectively. Abnormal feeding patterns and behaviour were not observed among the 4 groups during lactation. We found there was no significant difference in body weight among the low-protein diet and control groups 3 weeks after gestation (Table 1). Similar results were also observed for organs, i.e.; kidney weights in all groups. However, a previous report showed that body weight in the low-protein group was significantly increased when compared with the control group 23 weeks after gestation [24].

**Table 1:** Body weight and organ weight of female offspring at week 3.

Group	CC	LP	LPCL	LPCH
Body weight (g)	60.49±4.26	60.62±2.42	56.33±6.22	54.59±4.85
Kidney weight (g)	0.643±0.048	0.612±0.034	0.574±0.080	0.548±0.049
Kidney weight/BW	1.06%±0.05%	1.01%±0.04%	1.02%±0.05%	1.00%±0.02%

Values are means ± S.E.M. (n = 5–8). BW, body weight; CC, control on control; LP, control on protein restricted; LPCL, 0.12% GTE diet on protein restricted; LPCH, 0.24% GTE diet on protein restricted.

### Offspring plasma parameters

As listed in Table 2, total cholesterol (T-cho) in low-protein group administered with high dose of GTE was significantly increased when compared with the low-protein group. In addition, although insulin concentration in low-protein group was about 2 times higher than other groups, no significant difference was observed among all groups (Table 2).

**Table 2:** Plasma parameters of male and female offspring at week 3.

Group	CC	LP	LPCL	LPCH
T-cho (mg/dL)	80.7±6.6	71.7±9.2	92.1±4.5	96.3±3.2^b^
BUN (mg/dL)	9.96±0.91	9.12±0.67	9.92±0.66	9.66±0.82
CRE(mg/dL)	0.73±0.02	0.77±0.03	0.85±0.03	0.82±0.04
Na (mEq/L)	142.6±0.6	141.6±0.7	142.0±0.5	140.3±0.9
K (mEq/L)	2.66±0.41	2.86±0.32	2.91±0.19	2.89±0.12
Insulin (ng/mL)	0.348±0.093	0.812±0.518	0.381±0.097	0.459±0.101

Values are means ± S.E.M. (n = 5–8). T-cho, total cholesterol; BUN, blood urea nitrogen; CRE, creatinine; CC, control on control; LP, control on protein restricted; LPCL, 0.12% GTE diet on protein restricted; LPCH, 0.24% GTE diet on protein restricted. ^b^ *P* < 0.05 compared with LP.

### DNMTs expression in kidneys from 3 week old offspring

**Figure 2:**
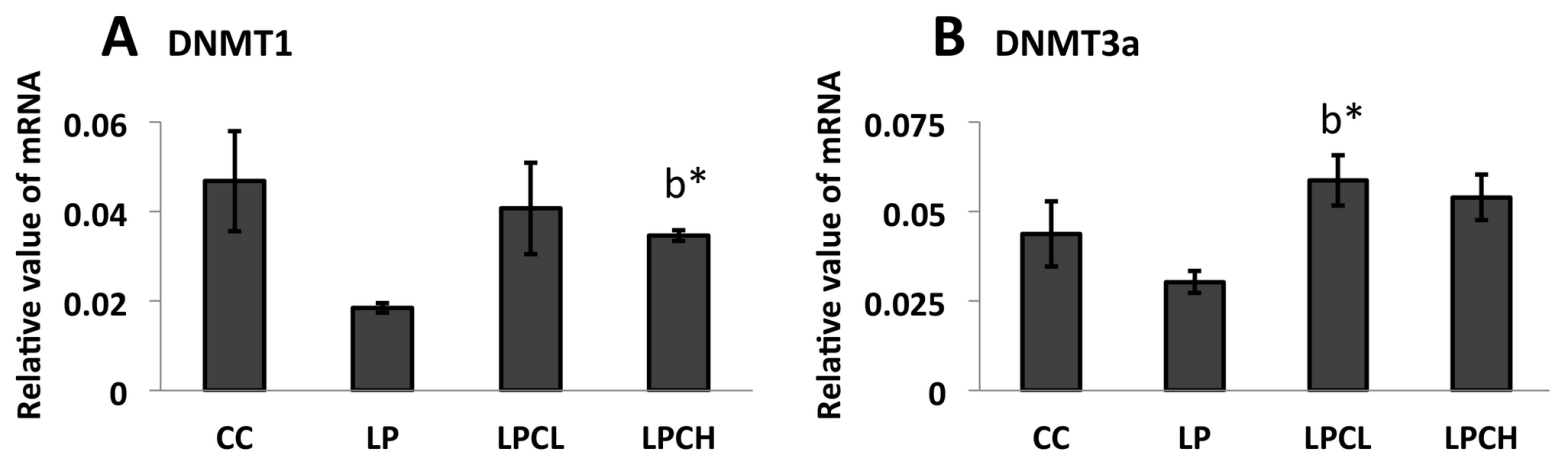
mRNA expression of DNMT1 and DNMT3a by real-time RT-PCR. **A**) DNMT1 in the kidneys of 3 week old postnatal offspring, and **B**) DNMT3a in the kidneys of 3 week old postnatal offspring. Values are means ± S.E.M. (n = 3; error bars indicate S.E.M.). CC, control on control; LP, control on protein restricted; LPCL, 0.12% GTE diet on protein restricted; LPCH, 0.24% GTE diet on protein restricted. ^b*^ means P<0.05 against LP.

To confirm whether enzymes which modify epigenetic marks were altered under different regimens of maternal nourishment, DNMT1 and 3a were measured in kidneys from offspring of undernourished pregnant rats with or without GTE treatment. As shown in Figure 2A, DNMT1 expression in the low-protein (LP) group was lower than the control group; however, there was no significant difference between these groups. By contrast, significant change was observed in the LPCH group compared to the LP group. These results indicated that GTE treatments were similar to the control. Similar patterns in LP and LPCL groups were observed in the case of DNMT3a (Figure 2B). In addition, there is no significant difference of DNMT3b expression among all groups (data not shown).

### SIRTs expression in kidneys from 3 week old offspring

**Figure 3:**
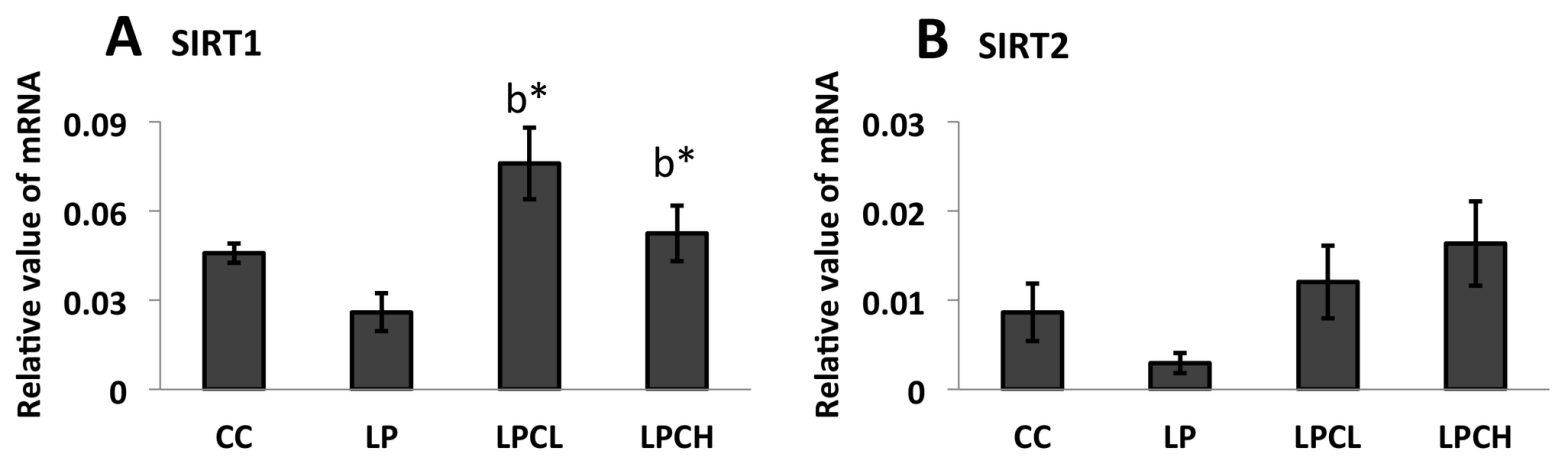
mRNA expression of SIRT1 and SIRT2 by real-time RT-PCR. **A**) SIRT1 in the kidneys of 3 week old postnatal offspring, and **B**) SIRT2 in the kidneys of 3 week old postnatal offspring. Values are means ± S.E.M. (n = 3; error bars indicate S.E.M.). CC, control on control; LP, control on protein restricted; LPCL, 0.12% GTE diet on protein restricted; LPCH, 0.24% GTE diet on protein restricted. ^b*^ means P<0.05 against LP.

**Figure 4:**
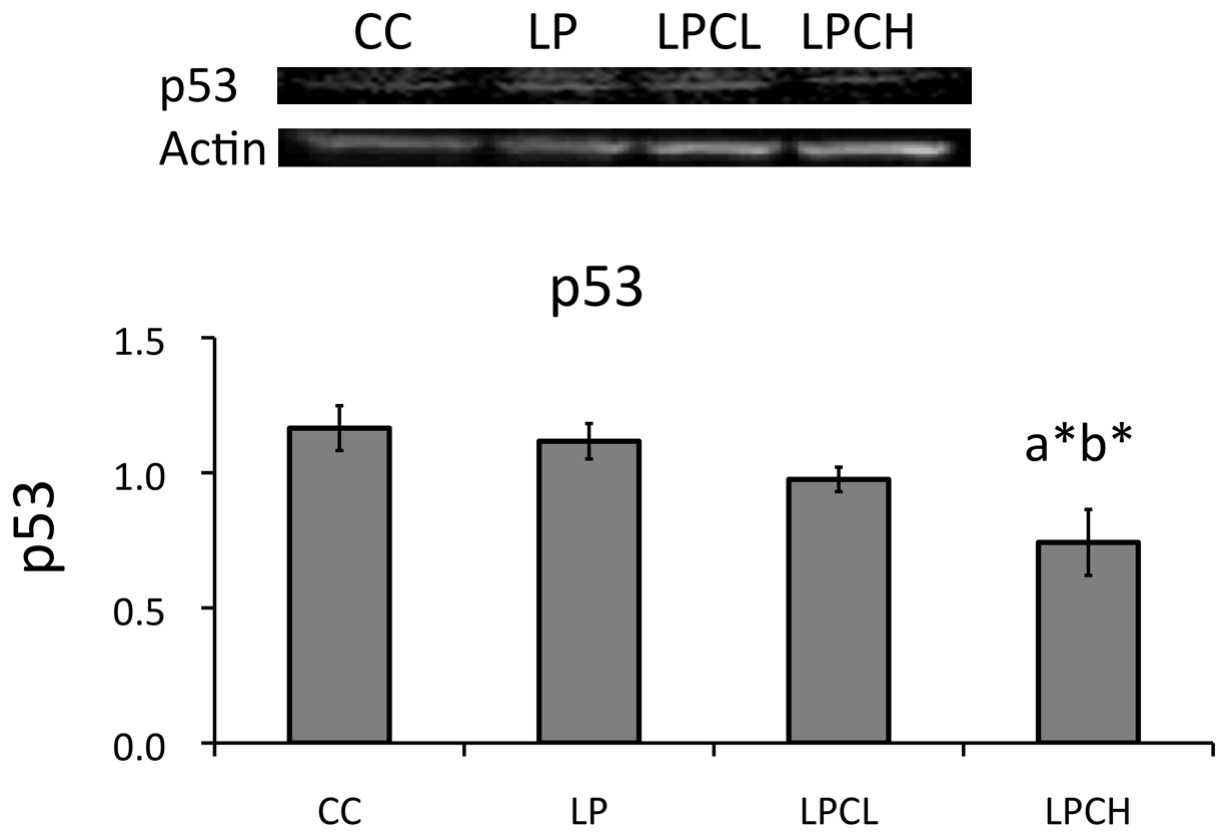
Protein abundance of p53 to β-actin in the kidneys of 3 week old postnatal offspring using western blot analysis. Values are expressed as means ± S.E.M. (n = 4). CC, control on control; LP, control on protein restricted; LPCL, 0.12% GTE diet on protein restricted; LPCH, 0.24% GTE diet on protein restricted. ^a*^P < 0.05 compared with CC. ^b*^P < 0.05 compared with LP.

To test if longevity related gene expression was sensitive to different regimens of maternal nourishment, SIRT1 and 2 were examined in kidneys from offspring of undernourished pregnant rats with or without GTE addition. Figure 3A shows that SIRT1 expression in low-protein group shows a tendency to decrease (p=0.0507) when compared with the control group. By contrast, GTE treatments were similar to the control (for example p=0.54 for control and high GTE treated groups). Similar patterns were observed in the case of SIRT2 (Figure 3B). In detail, p values in SIRT2 gene expression are 0.091 for low protein group and low GTE treated group, and 0.099 for low protein group and high GTE treated group. To investigate changes of downstream of SIRT1, p53 contents were detected in kidneys from offspring of undernourished pregnant rats with or without GTE addition using western blotting (Figure 4). Unexpectedly, p53 was significantly downregulated in the kidney of high dose GTE treated low protein group compared to the un-treated low protein group.

### AMPK phosphorylation in kidneys from 3 week old offspring

**Figure 5:**
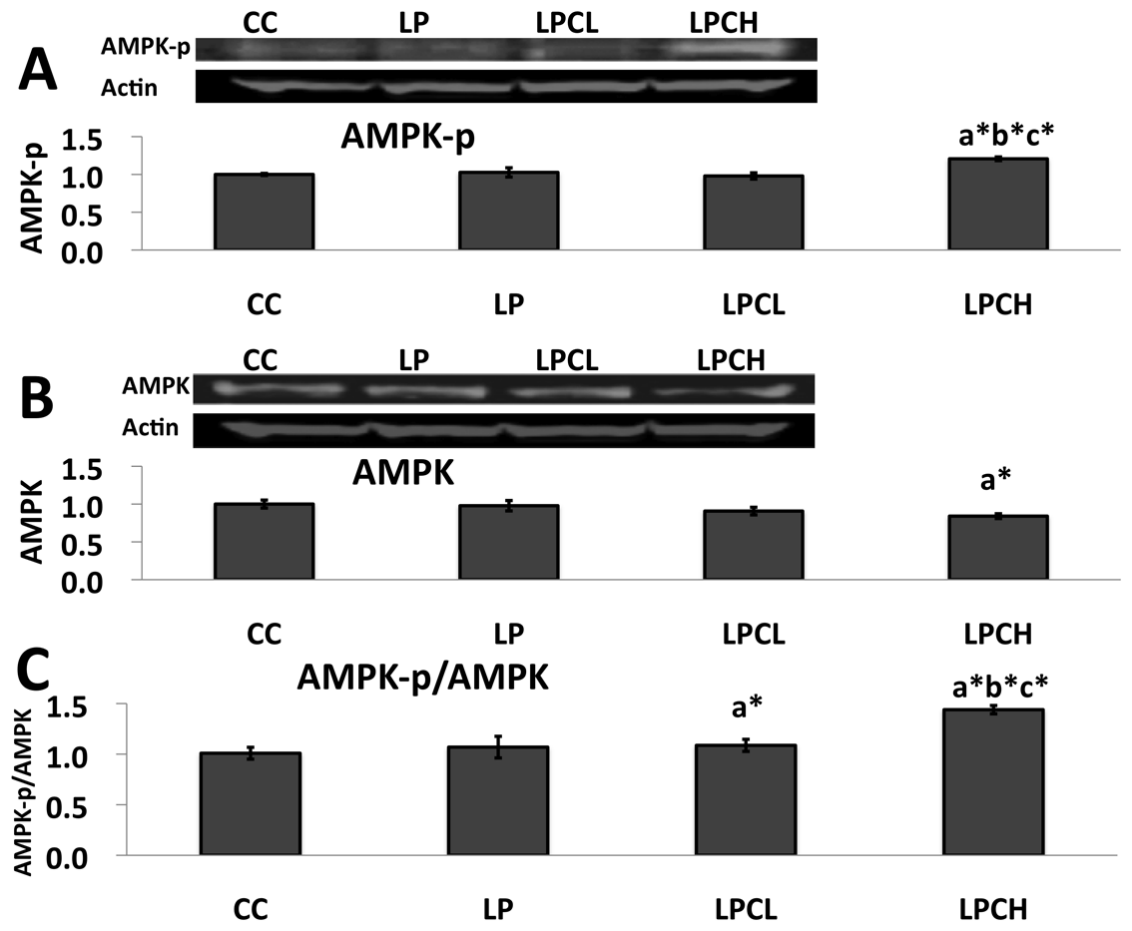
Protein abundance of AMPK in the kidneys of 3 week old postnatal offspring using western blot analysis. **A**) phosphorylated AMPK to β-actin, **B**) AMPK to β-actin and **C**) phosphorylated AMPK to total AMPK. Values are expressed as means ± S.E.M. (n = 4-7). CC, control on control; LP, control on protein restricted; LPCL, 0.12% GTE diet on protein restricted; LPCH, 0.24% GTE diet on protein restricted; and AMPK-p, phosphorylated AMPK. ^a*^P < 0.05 compared with CC. ^b*^P < 0.05 compared with LP. ^c*^P < 0.05 compared with LPCL.

To detect lipid-metabolism in kidneys of newborns from the low-protein group, total and phosphorylated AMPKs were measured in kidneys from all groups by western blotting. As shown in Figure 5, phosphorylated AMPK expression in kidneys from low-protein group treated with high GTE dose was higher than in the low-protein group. This difference markedly increased when phosphorylated AMPK to AMPK ratio was considered (Figure 5C).

### eNOS phosphorylation in kidneys from 3 week old offspring

**Figure 6:**
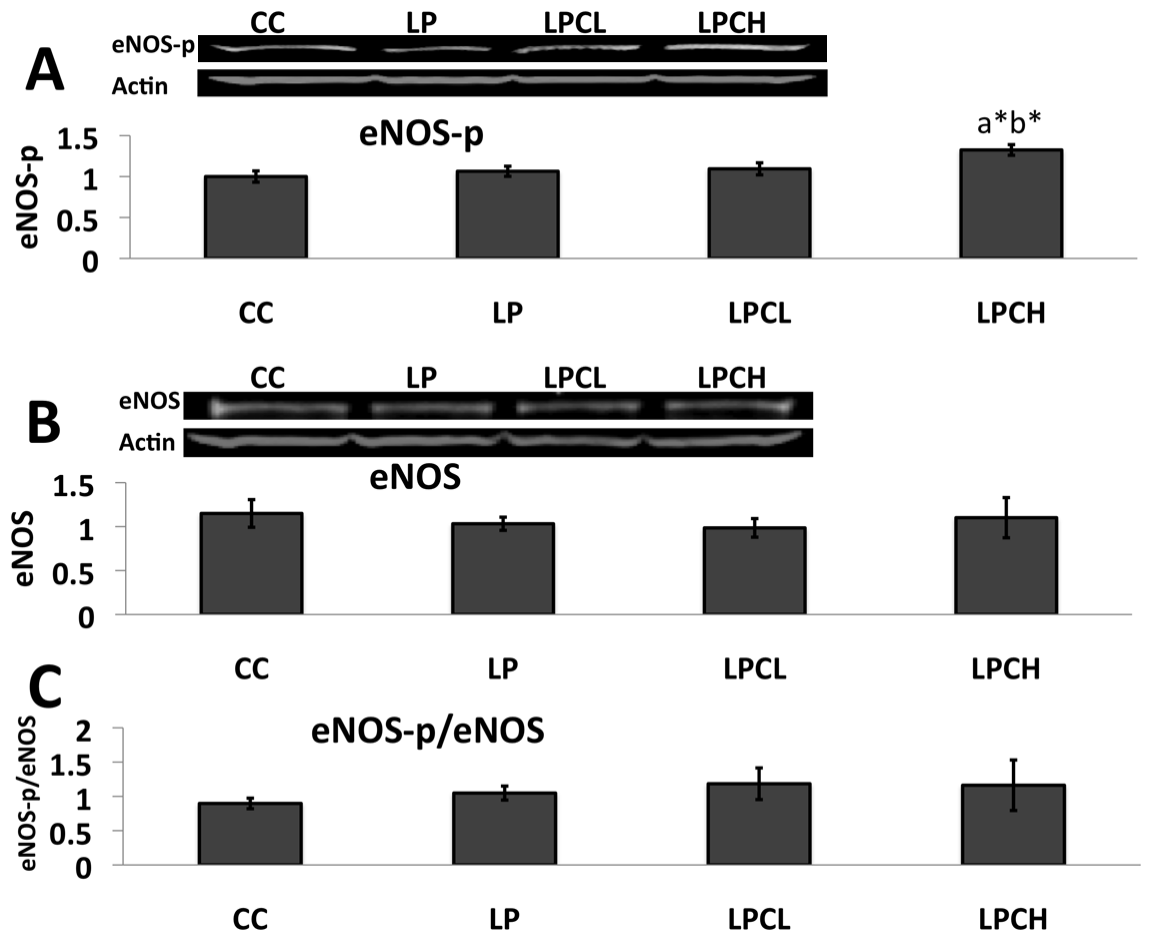
Protein abundance of eNOS in the kidneys of 3 week old postnatal offspring using western blot analysis. **A**) phosphorylated eNOS to β-actin, **B**) eNOS to β-actin and **C**) phosphorylated eNOS to total eNOS. Values are expressed as means ± S.E.M. (n = 4-7). eNOS-p, phosphorylated eNOS; CC, control on control; LP, control on protein restricted; LPCL, 0.12% GTE diet on protein restricted; LPCH, 0.24% GTE diet on protein restricted. ^a*^P < 0.05 compared with CC. ^b*^P < 0.05 compared with LP.

Because AMPK is reported to be associated with the phosphorylation of eNOS, resulting in an increased release of NO [27,28], we investigated the expression and phosphorylation of eNOS. Although phosphorylated eNOS in the of low-protein group with high GTE dose significantly increased when compared with the control group, phosphorylated eNOS among groups was not different in low-protein groups (Figure 6A). As shown in Figure 6B and C, no significant difference was found, respectively, for eNOS and phosphorylated eNOS /eNOS ratio among all groups.

## Discussion

It has been well known that low-protein diet during pregnancy affects blood pressure and renal function after maturation [29]. Many epidemiological and experimental studies have revealed that fetal and neonatal nutritional conditions are highly associated with subsequent dysfunctional development in life. For example, fetal malnutrition was an early-life inducer of glucose intolerance and obesity [30–32]. In contrast, body weight of female offspring in this study (Table 1), and male offspring (both age of 3 weeks, maternal low-protein diet in pregnancy) have no change when compared with controls [24], although body weight of 23 week old offspring from undernourished rats is significantly heavier than controls [24]. However, there is little research about interaction between lipid-metabolism and enzymes which modify epigenetic marks in offspring kidneys from dams with low-protein diet. We confirmed whether enzymes which modify epigenetic marks were altered under different regimens of maternal nourishment using real-time RT-PCR technique. In this study, we demonstrated that DNMT1, DNMT3a, SIRT1 and SIRT2 in kidneys of newborns from low-protein diet mothers showed a decrease in gene expression when compared to the control. In addition, the decrease was reversed in GTE treatments, also accompanied by an increase of phosphorylated AMPK. From these results, it was suggested that further research be done to investigate whether these changes in enzyme expression result in any changes in epigenetic modifications and renal energy metabolism. Isbrucker et al. [22] found that epigallocatechin gallate (EGCG) from green tea during organogenesis was neither toxic nor teratogenic to dams and fetuses from pregnant rats fed catechin (1200-2400 ppm, i.e.; 0.12-0.24%) supplemented diets.

DNMT1 has been reported to work mainly embryonic development to maintain methylation through mitosis [16]. DNMT3a has been proposed to play a role in gene expression regulation by promoting *de novo* methylation for gene silencing [17]. In this study, DNMT1 and DNMT3a gene expression in GTE treatments was significantly up regulated when compared with the low-protein control group. Thus, our results showed that during the lactation period DNMT1 gene expression in the kidney of low protein offspring had a tendency to decrease (p=0.066) because of undernourishment during pregnancy, but this difference was not observed when GTE supplemented low protein diets (p=0.71 and 0.34 for control group and low or high GTE treated group, respectively). It has been established that fetal malnutrition is an early-life inducer of glucose intolerance and obesity [30,32]. Furthermore, *de novo* synthesis depending on DNMT3a was activated by GTE addition. It was suggested that further research could investigate the impact of the gene expression changes in GTE treatment on obesity and DNA methylation. However, Li and Tollefsbol [33] reported that catechin inhibited DNMT1 protein activity by binding itself to the DNMT1 catalytic center. From their results, it was suggested if DNMT1 activity was inhibited when excessive amounts of DNMT1 were expressed in the cells.

It has been reported that in vitro exposure to ECG, a kind of tea catechin, induces apoptosis and retards early post-implantation development after mouse blastocysts are transferred to host mice [34]. Our previous research [26] showed that DNMT3a gene expression decreased in the case of apoptosis induced by serum deprivation in PC12 cells. In this study, DNMT3a gene expression in kidneys from 3 week old offspring was increased by GTE addition.

Peng et al. [15] proposed cooperation of SIRT1 and DNMT1 in MDA-MB-231 breast cancer cells. We also reported that DNMT1 and DNMT3a have a close relationship with SIRT1 [26]. In this study, SIRT1 and SIRT2 gene expression in low-protein control and normal control was not significantly different. Similar results were obtained for DNMT1 and DNMT3a gene expression (Figure 2). Recently, Fullerton and Steinberg [35] reported that SIRT1 played a role on AMPK in the modulation of insulin sensitivity by resveratrol. Price et al. [36] indicated that SIRT1 was required for AMPK activation. To estimate the phosphorylation state of AMPK, which is a major regulator in lipid metabolism, total and phosphorylated AMPK protein levels in kidneys of young offspring were analyzed by western blotting (Figure 5). A similar pattern was observed between amounts of phosphorylated AMPK in the kidney of 3 week old female and in liver of 3 week old male animals. However, no significant difference in phosphorylated AMPK levels was seen in newborns from low-protein and control rats. Diets with polyphenols, such as catechin in this study or azuki bean coat-derived polyphenol added to low-protein regimens during pregnancy increased the amount of phosphorylated AMPK in both female and male 3 weeks after birth. In this study, SIRT1 expression in kidneys of high and low dose GTE treatment groups was significantly increased when AMPK was activated by GTE (Figure 3A). On the other hand, SIRT2 gene expression was not significantly affected (Figure 3B).

Thors et al. [37] reported that activation of AMPK contributes to eNOS phosphorylation. In addition, Sato et al. [38] found that phosphorylated eNOS in livers of 23 week old postnatal offspring from low-protein and quercetin dams was significantly increased when compared with controls. Therefore, we examined the expression and phosphorylation of eNOS. In our study, the expression of phosphorylated eNOS in kidneys of female offspring from lactating rats with high GTE dose treatment was significantly increased when compared with low-protein diet control. However, phosphorylated eNOS-p/eNOS level was not significantly different (Figure 6). These results were in agreement with phosphorylated eNOS in livers of 3 week old offspring from dams with low-protein and quercetin diet [38]. The reason why phosphorylated eNOS did not differ on treatment with GTE when compared with the control group is still unclear, despite increased phosphorylated AMPK (Figures 2 and 3). However, this phenomenon may depend on offspring age because phosphorylated eNOS in offspring from low-protein and quercetin treatment dams was increased significantly when compared with the control group [38]. Kang et al. [39] reported that phosphorylated eNOS increased depending on age during juvenile growth. In addition, the increase of phosphorylated eNOS in kidneys of the high GTE dose treatment group was observed. It was also reported that inhibition of SIRT2 down-regulates eNOS level leading to premature senescence-like phenotype in endothelial cells [40,41].

In conclusion, we show here that GTE intake during lactation up-regulates the activation of AMPK in young female offspring from protein-restricted dams and modulates the AMPK pathway in the kidney. This pathway may be stimulated by SIRT1 gene expression. Although the reasons for the effects of GTE intake during lactation on the AMPK pathway in the kidney remain unclear, the feeding of GTE, an AMPK activator, to protein-restricted dams during lactation is likely to up-regulate AMPK activation and may partly lead to alterations of AMPK pathway in kidneys of female offspring from protein-restricted dams. In addition, energy metabolism in fetal and offspring period with GTE addition might be related enzymes which modify epigenetic marks such as DNMT1 and DNMT3a.
